# Development of Harlequin Syndrome following placement of thoracic epidural anesthesia in a pediatric patient undergoing Nuss procedure

**DOI:** 10.1002/ccr3.1097

**Published:** 2017-08-03

**Authors:** Ashley Lefevre, Gregory Schnepper

**Affiliations:** ^1^ Department of Anesthesiology Children's Healthcare of Atlanta Emory University Atlanta Georgia; ^2^ Department of Anesthesia & Perioperative Medicine Medical University of South Carolina Charleston South Carolina

**Keywords:** Harlequin Syndrome, Nuss procedure, pectus excavatum, thoracic epidural, unilateral facial flushing

## Abstract

We report the development of Harlequin Syndrome following thoracic epidural placement in a pediatric patient. Unilateral facial flushing with contralateral pallor and anhidrosis is the clinical presentation. This syndrome is typically benign. When related to regional anesthesia, treatment involves reducing the local anesthetic infusion or stopping it altogether.

## Graphical abstract

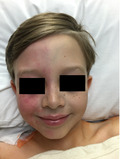

## Introduction

A thoracic epidural is a common pain management option for patients undergoing a Nuss procedure for pectus excavatum. We report a case of Harlequin Syndrome following placement of a T4‐5 epidural. Harlequin Syndrome is generally benign and believed to be caused by sympathetic interruption of the T2‐T3 vasomotor and sudomotor fibers. If the syndrome is related to regional anesthesia, decreasing or stopping the local anesthetic infusion should resolve symptoms. Reassurance to the patient, family, and healthcare providers is also important.

## Case Description

A 10‐year‐old, 30 kg male with pectus excavatum presented to the operating room to undergo a Nuss procedure. The patient reported mild dyspnea on exertion but was otherwise healthy. Following inhalational induction with sevoflurane, IV placement, and securement of an endotracheal tube, the patient was positioned in the left lateral decubitus position for epidural placement.

The thoracic epidural space at T4‐5 was identified using a 17 gauge Tuohy needle by a loss of resistance (LOR) technique with saline. The LOR was obtained at 4 cm, and a 19 gauge catheter was secured at 7 cm to the skin. Following a negative 3 mL test dose of 1.5% lidocaine with 1:200,000 epinephrine for intravascular and intrathecal placement, the patient received a 3 mL bolus of 0.2% ropivicaine. An infusion of 0.2% ropivicaine with 5 mcg/mL of hydromorphone and 2 mcg/mL of clonidine was started at 6 mL/h and continued during the case and into recovery. The surgery was completed without complication. Two hours after the initiation of the epidural and prior to emergence the patient received an additional 3 mL bolus of the epidural solution. The patient was extubated and transported to the recovery room without event.

**Figure 1:**
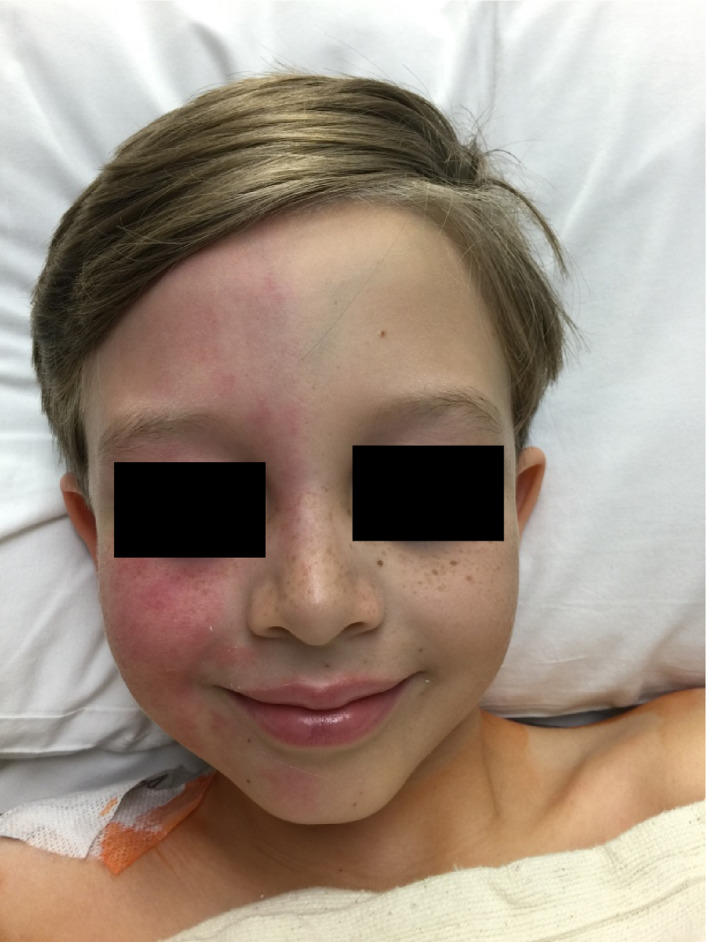
Unilateral facial flushing and contralateral facial pallor with sharp midline demarcation.

Three hours after arrival to the recovery room, the patient's nurse paged the pediatric pain service with concerns about the striking redness on the right side of the patient's face. Examination revealed unilateral facial flushing on the right, with a sharp midline demarcation (Fig. 1). The patient's contralateral side demonstrated pallor and anhidrosis. The patient also reported a paresthesia in his left hand and fingertips. The paresthesia was located in a C7 and C8 dermatomal distribution. The remainder of the neurologic exam was normal. The epidural solution was decreased to 4 mL/h, and he was kept under close observation. The only medication the patient received in the recovery room was 0.2 mg of IV hydromorphone approximately 2 h prior to the flushing.

Within 4–5 h the patient had complete resolution of the left hand paresthesia, facial flushing, and pallor. The epidural infusion was continued at 4 mL/h. The pediatric pain service continued to follow him during his hospital stay. The remainder of the patient's stay was uneventful. The epidural catheter was removed on POD#3 without event, and the patient was discharged on POD#4.

## Discussion

The sudden appearance of unilateral facial flushing can be dramatic and unnerving to patients, families, and healthcare providers. “Harlequin Syndrome” was coined by Lance 1 to describe this sign. Harlequin Syndrome is a rare neurological condition that results in the appearance of unilateral facial flushing. The contralateral side demonstrates pallor and anhidrosis and is the physiologically disturbed side. It is believed to occur from blockade of T2‐T3 sympathetic vasodilator and sudomotor nerves. This disruption leads to unilateral facial pallor and anhidrosis. The neurologically intact side appears flushed. It is not clear whether the flushing and sweating are a normal or excessive response on the side with the intact sympathetic innervation. The unilateral flushing and sweating could be an exaggerated release phenomenon, compensating for the loss of sweating and flushing on the side with the sympathetic deficit 1.

**Figure 2:**
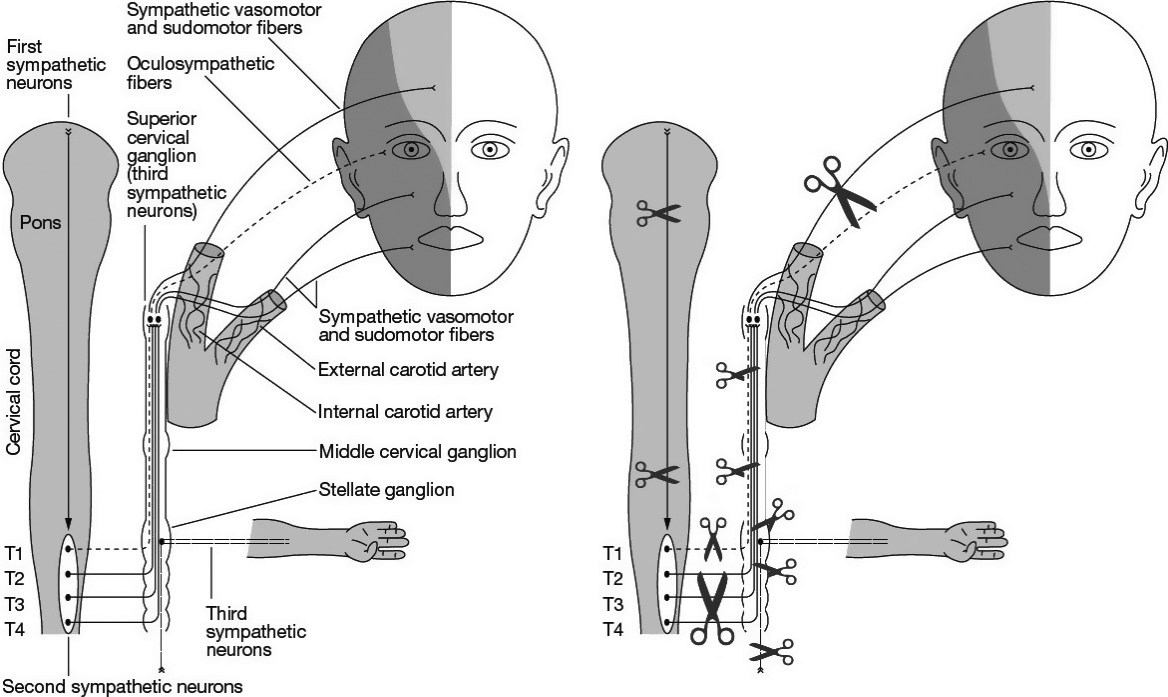
Sympathetic fibers innervating the face, and possible lesion sites as indicated by scissors. Harlequin syndrome is believed to be caused by interruption of the T2‐T3 vasomotor and sudomotor fibers which could occur due to various causes at any of these locations 2.

Additionally, involvement of T1 fibers is possible resulting in Horner syndrome. Figure 2 displays this relationship and possible locations of a lesion which may lead to Harlequin Syndrome. The symptoms may be triggered by thermal, emotional, or exertional stimuli 1, 2. Unilateral facial flushing may also be a sign of underlying malignancy such as a Pancoast tumor, superior mediastinal neurinoma, or schwannoma, but most cases of Harlequin Syndrome are benign 2. If a structural lesion has been excluded the probable diagnosis is a restricted autonomic neuropathy with a benign prognosis.

Harlequin Syndrome has been reported to occur following regional anesthesia including thoracic epidural 3 and paravertebral 4 blocks. It has been rarely reported in the pediatric population including following neck dissection 5 and regional anesthesia 3.

The authors believe this patient developed Harlequin Syndrome secondary to regional anesthesia. The level of placement of a thoracic epidural in a patient undergoing a Nuss procedure will be dependent on the anesthesia provider but will typically be placed at a T4/5 to T6/7 level. Once the epidural space is accessed, a catheter is threaded into the epidural space. It is unknown where the catheter will travel as it is threaded into the epidural space. In the adult population, it has been suggested to leave 5 cm of the catheter in the epidural space to limit the possibility of the catheter either mi'grating out of the epidural space or increasing the possibility of a unilateral block and transforaminal escape 6. For the pediatric population, there is not a consensus for how much length a catheter should be left in the epidural space, but it is generally less (~3 cm) than adults due to the smaller anatomic space. Because our patient's epidural was placed at a high level (T4/5), it is likely the catheter migrated cephalad and unilaterally to the left. The authors hypothesize that although the patient received an appropriate volume of local anesthetic through the epidural by bolus and infusion, when combined with a migrated catheter conditions were created that led to the Harlequin appearance. Once the patient's epidural infusion was decreased, the Harlequin appearance disappeared. Our patient also demonstrated a left upper extremity paresthesia in a C7‐8 dermatomal distribution. This was likely due to the high spread of the local anesthetic affecting the C7‐8 nerve roots unilaterally. Once the local anesthetic infusion was decreased this paresthesia also resolved.

Complete resolution of Harlequin Syndrome commonly occurs if appropriate treatment is initiated. If regional anesthesia is the trigger, treatment is decreasing the local anesthetic infusion or stopping it altogether, combined with close observation of the patient. Reassurance to the patient, family, and other healthcare providers is also important due to the anxiety involved from the dramatic and colorful presentation.

## Conflict of Interests

Published with written consent of parents. No conflict of interests declared. No funding required.

## Authorship

AL and GS: all contributed to the preparation, review, and submission of this manuscript.
